# Reliability and validity of the brief version of the difficulties in emotion regulation scale in a sample of Turkish adolescents

**DOI:** 10.1186/s40359-023-01199-y

**Published:** 2023-05-19

**Authors:** Dilara Demirpence Secinti, Ezgi Sen

**Affiliations:** Şişli Etfal Research and Training Hospital, Child and Adolescent Mental Health, Istanbul, Turkey

**Keywords:** DERS-16, Reliability, Validity, Adolescents

## Abstract

**Background:**

Difficulty in regulating emotions increases during adolescence and can be associated with psychopathology. It is thus crucial to develop tools to identify adolescents at risk of having emotional difficulties. This study aimed to investigate the reliability and validity of a brief questionnaire in a sample of Turkish adolescents.

**Methods:**

A total of 256 participants (mean age = 15.51 ± 0.85) were recruited. They completed the original form of the Difficulties in Emotion Regulation Scale (DERS-36), a brief version of DERS (DERS-16), the Barrett Impulsivity Scale (BIS-11), and the Toronto Alexithymia Scale (TAS). Psychometric properties of DERS-16 were investigated by confirmatory factor analysis, Cronbach’s alpha, and Pearson correlational analysis.

**Results:**

A five-factor model and second-order bifactor model of DERS-16 were confirmed. Cronbach’s alpha values for the subscales varied between 0.69 and 0.88, while the reliability of the factors Difficulties in Emotional Processing and Difficulties in Emotion Regulation were 0.75 and 0.90, respectively. DERS-16 subscales were positively correlated with the BIS-11 and TAS. In addition, there were only minimal differences between the DERS-16 and DERS-36.

**Conclusion:**

The DERS-16 is a valid and reliable scale for Turkish adolescents. The fact that it has fewer items than DERS-36, but has similar reliability and validity and can be used as two factors, provides significant advantages in terms of applicability.

## Introduction

Emotion regulation (ER) is an essential process for mental and physical well-being. Research in ER shows that ER deficits are associated with a wide range of mental health problems [1]. The effort expended on ER is greater in adolescence than at other ages, especially in late adolescence as the use of ER strategies decreases during this time [2]. Thus, the ability to determine individual differences in ER and possible associated factors is crucial to detect those at risk to prevent possible psychiatric disorders. ER consists of three parts: the process of perceiving and identifying emotions (perception), the evaluation and selection of the required regulation (valuation), and the implementation of the appropriate behavior, namely action [3]. In other words, after recognizing and understanding the emotion in response to a trigger, reappraisal or distraction could be selected, according to the severity and type of the experienced emotion. The third step is to act. Difficulties experienced in any of these three steps are defined as ER difficulties. Considering these three steps, perception, the first part of emotion regulation, has common features with alexithymia, which is associated with difficulty in identifying and expressing emotions, and difficulty in distinguishing bodily sensations from emotions, in response to emotional stimuli [4]. In the last stage of ER, people act by choosing the appropriate strategy based on the emotions they feel. However, some individuals act without choosing a healthy strategy, which is defined as impulsivity [5]. Studies in the literature show that difficulties in ER are associated with impulsivity and alexithymia [6, 7].

There are several empirically supported measures of various dimensions of ER difficulties. A prominent example is the Difficulties in Emotion Regulation Scale (DERS), a theoretically driven, self-report measure that is used in many languages [8]. DERS includes 36 items that assess typical levels of difficulties in ER. It has a multidimensional construct including the ability to be aware of, understand, and accept emotions; the capability to inhibit impulsive behaviors under the influence of negative emotions; flexible use of strategies, appropriate to the situation, to modulate the intensity and/or duration of emotions to act in line with individual goals; and willingness to experience negative emotions while pursuing personally meaningful life activities [9]. The original version of the DERS scale consists of six subscales (Awareness, Nonacceptance, Clarity, Goals, Impulse, and Strategy). It is thought that the Awareness, Nonacceptance, and Clarity subscales evaluate difficulty in emotional processing (DEP), the Goals and Impulsivity subscales evaluate difficulty in emotional response (DER), and the Strategy subscale includes emotional self-efficacy [10]. For this reason, the literature includes the second-order bifactors model, and six-factor and four-factor uses of the DERS-36 scale, in which the Strategy and Awareness subscales are extracted.

However, although it is comprehensive, a scale consisting of 36 items may be challenging to administer in some situations or settings [11]. To have broad clinical and research utility, a scale needs to be short enough to use in clinics with limited time resources, in studies that involve assessments with tight intervals (e.g., weekly administration), and in large epidemiological studies [1]. It is also known that people are less eager to complete longer scales [12]. There is therefore a need for a briefer version of the DERS. Furthermore, previous studies have shown that the Awareness subscale has lower correlations with the other DERS subscales, suggesting that it might not be measuring the same underlying construct. Thus, Bjureberg et al. [8] generated a 16-item version of the scale (DERS-16), reducing the number of items and removing the awareness subscale. In their study, two versions of the DERS were significantly correlated with other scales measuring emotional functioning, psychiatric symptoms, self-harm, and suicidal thoughts. In the Bjubergs study [11], he validity and reliability of the DERS-16 subscales were not studied, and the validity and reliability of the scale were assessed by calculating a single factor over the total score. The goodness of fit indices of single-factor, second-order bi-factor, and five-factor models were investigated for both DERS-16 and DERS-36 [13, 14]. However, the five-factor model was most frequently used in the literature [15, 14]. In addition, studies investigating the structural validity of the five-factor DERS-16 scale report that its subscales were reliable [14]. In one study, the correlations between the subscales of DERS-16 and DERS-36 ranged between 0.04 and 0.85, and 0.14 and 0.90, respectively [16]. Thus, studies of the reliability and validity of DERS-16 conclude that it is not substantially different from DERS-36 and may be preferred due to its lower number of items and greater ease of application.

The Turkish version of the DERS-16 has been developed and tested only in adult samples [14] and has not been studied in adolescents. However, it is known that ER and emotional processing change with age [2]. There are differences in ER, at both the neurobiological and behavioral levels, between middle adolescence and late adolescence, and even between late adolescence and emerging adulthood [17]. There is therefore a need for the validity and reliability of the DERS-16 scale to be tested in Turkish adolescents. Thus, this study aimed to examine the psychometric properties of DERS-16 in this population. Hence, the five and single-factor models, and the second-order bifactor model of DERS-16 were examined using confirmatory factor analysis (CFA). In the second stage of the study, to show construct validity, the correlations between the BIS-11, Toronto Alexithymia, DERS-16, and DERS-36 scales were investigated.

## Method

### Study design and participants

The baseline data was collected between December 2020 and June 2021 via an online Google Documents application, using convenience sampling. The link to the study was shared in WhatsApp groups with high school teachers living in Gaziantep, Kilis, and Istanbul. These teachers shared the link in WhatsApp groups used by themselves and the parents of high school students. Informed consent was obtained from the parents before participation. After parental consent was obtained, the questionnaire was distributed to the adolescents through their parents. All the adolescents were in public schools. The inclusion criteria of the study were that participants should be aged 14–18 and a student in high school. Ethical approval was obtained from the Ethics Committee of Rumeli University.

### Instruments

Baseline demographic characteristics (age, gender, educational status of parents, city, city district, and any psychiatric and medical disorders of adolescents) were recorded in a socio-demographic data form (Table 1). The socioeconomic status of the families and ethnicity of adolescents were not obtained directly.

In total, 256 participants (50% female) were recruited. The mean age was 15.51 ± 0.85. Most parents (34.95%) graduated from high school. Only 2.7% of adolescents had a psychiatric diagnosis and 3.9% had a physical medical disorder. Three participants stated that they had an anxiety disorder, one that he/she had obsessive-compulsive disorder, and a further three that they had psychiatric disorders but these were not specified. There were no missing data.

**Table 1:** Demographic characteristics of the participants

Variables	N	% of Total Sample
*Gender*		
Female	128	50
Male	128	50
*Education of the Fathers*		
Primary school	101	39.4
High school	87	34
University	68	26.6
*Education of the Mothers*		
Primary school	110	43
High school	92	35.9
University	54	21.1
*Psychiatric Diagnosis*		
Yes	7	2.7
No	249	97.3
*Physical Medical Disorder*		
Yes	10	3.9
No	246	96.1

### The brief version of the difficulties in emotion regulation scale (DERS-16)

The questionnaire was developed after shortening the original DERS form [8]. It consists of 16 items rated on a 5-point positively valenced Likert Scale, with subscales measuring nonacceptance of negative emotions (Nonacceptance, three items), inability to engage in goal-directed behaviors when distressed (Goal, three items), difficulties controlling impulsive behaviors (Impulsivity, 3 items), limited access to emotion regulation strategies perceived as effective (Strategy, five items), and lack of emotional clarity (Clarity, two items). The DERS-16 has been shown to have satisfactory psychometric properties [8], similar to those of the original DERS. Subscale scores are calculated as the total score of their respective scale items and the total DERS-16 score is the total of all the subscale scores.

Before the translation process, we obtained permission to translate the English version of DERS-16 from the corresponding author in Bjureberg et al.’s [8] original study. The translation process of DERS-16 was carried out in five stages: initial translation by two child psychiatrists working independently, comparison of the two translations and transformation of these to a single final version, back translation of the final version by a third translator, administering the final version to focus groups that included 10 adolescent volunteers, and final revision of the translation.

### Difficulties in emotion regulation (DERS)

This scale was developed by Gratz-Roomer [9] and consisted of 36 items and 6 subscales, namely nonacceptance of emotions (Nonacceptance), inability to enact goal-directed behavior under negative circumstances (Goal), difficulties in impulse control (Impulsivity), non-awareness of negative emotions (Non-Awareness), limited access to ER strategies (Strategy), and clarity in emotional reactions (Clarity). The sum of the subscales gives the total score.

The internal consistency for each DERS subscale in the current sample was very good: Nonacceptance (α = 0.93), Goal, (α = 0.90), Impulsivity, (α = 0.90), Nonawareness (α = 0.93), Strategy (α = 0.77), Clarity (α = 0.87). The Cronbach’s alpha value for the whole scale was 0.92.

### Toronto alexithymia questionnaire

TAS was developed to assess difficulty in identifying feelings, difficulty describing feelings, and externally oriented thinking [18]. It is comprised of 20 items and is rated on a 5-point Likert scale ranging from 1 (strongly disagree) to 5 (strongly agree). Here, we used the Turkish version which is reliable and valid in Turkish samples [19]; it also showed good reliability (α = 0.81) in the current study.

Barratt Impulsiveness Scale-Short Form (BIS-11).

BIS-11 is a 15-item self-report questionnaire, rated on a 4-point scale [20]. It assesses impulsiveness under three areas following attention (inattention and cognitive instability), motor (motor impulsiveness and lack of perseverance), and non-planning (lack of self-control and intolerance of cognitive complexity). The sum of these subscales gives the total impulsivity score. In this study, Cronbach’s alpha was 0.83.

### Statistical analyses

Statistical analysis was conducted using IBM SPSS Statistics for Windows, Version 26.0, and PROCESS function V.2.16.1 in SPSS V.21 and AMOS. There were no missing data as all questions in the online survey required a response. *P*-values of less than 0.05 were regarded as statistically significant and exact *p*-values were reported to indicate the level of significance in the findings.

The first step of the five-factor model, the second-order bifactor model, and the one-factor model were analyzed using CFA. Kline [20] stated that the minimum fit indices of Chi-square, comparative fit index (CFI), root mean square error of approximation (RMSEA), and standardized root mean square residual (SRMR) should be reported in CFA analysis. In the literature, it has been reported that it is acceptable for the RMSEA value to be between 0.05 and 0.08 [21], the χ2/df value to be less than 3 [22], the goodness-of-fit index (GFI) and CFI values to be greater than 0.80 [23], and the SRMR value to be less than 0.80 [24].

For the second-order bifactor CFA, the Clarity and Nonacceptance subscales were analyzed under the DEP factor, while the Strategy, Impulsivity, and Goal subscales were analyzed under the DER factor. The reliability of DERS-16 was investigated using Cronbach’s alpha. The relationships between DERS-16, DERS-36, TAS, and BIS-11 were investigated with Pearson correlational analysis for concurrent validity.

## Results

### Confirmatory factor analysis

CFA analysis was performed for the five-factor model of the DERS-16 scale. This model 1emonstrated good fit: χ2 (94) = 239.658, RMSEA = 0.078, GFI = 0.895, CFI = 0.934, and SRMR = 0.067. The goodness-of-fit statistics of the second-order bifactor model were almost identical to those of the five-factor model 1: χ2 (98) = 248.409, RMSEA = 0.078, GFI = 0.891, CFI = 0.932, and SRMR = 0.055. CFA analysis of the 16-item single-factor DERS-16 scale was performed and it showed poor fit: χ2(104) = 732.768, RMSEA = 0.154, GFI = 0.720, CFI = 0.717, and SRMR = 0.089.

### Reliability analysis of DERS-16

The internal consistency (Cronbach’s α) of the DERS-16 was 0.92. The same values for the Clarity, Nonacceptance, Goal, Impulsivity, and Strategy subscales were 0.79, 0.69, 0.83, 0.86, and 0.88, respectively. Spearman-Brown coefficients of the Clarity, Nonacceptance, Goal, Impulsivity, and Strategy subscales were as follows: 0.79, 0.66, 0.78, 0.91, and 0.82, respectively.

In the second-order bifactor model, when the items of the Clarity and Nonacceptance scales were placed under the DEP factor, the reliability of this 5-item factor was 0.75; when the remaining items entered the analysis under the DER sub-factor, its reliability was 0.90.

### Concurrent and content validity of DERS-16

Table 2 shows the correlation between the subscales of the DERS-16 and DERS-36 scales, and Tables 3 and 4 show the correlations between these two scales and the TAS and BIS-11 scales. These demonstrate statistically significant correlations among the DERS-16 and DERS-36 subscales, and that they were also significantly correlated with the other two scales.

**Table 2:** Correlations between subscales of DERS-16 and the subscales of DERS-36

DERS-16 subscales	Subscales of DERS-36						
	DERS-C	DERS-G	DERS-I	DERS-S	DERS-N	DERS-A	DERS-T
Clarıty	0.75**	0.31**	0.53**	0.54**	0.39**	0.22**	0.64**
Goal	0.45**	0.82**	0.39**	0.51**	0.026**	-0.02	0.59**
Impulsivity	0.46**	0.33**	0.83**	0.58**	0.47**	0.13*	0.67**
Strategy	0.60**	0.46**	0.62**	0.84**	0.50**	0.16*	0.78**
Nona	0.44**	0.29**	0.47**	0.56**	0.80**	0.24**	0.68**
Total	0.68**	0.58**	0.74**	0.82**	0.62**	0.18**	0.87**
DEP	0.67**	0.38**	0.58**	0.65**	0.74**	0.27**	0.78**
DER	0.62**	0.61**	0.73**	0.80**	0.51**	0.12	0.83**

Note. DERS-C: Clarity subscale of DERS; DERS-G: Goal subscale of DERS; DERS-I: Impulsivity subscale of DERS; DERS-S: Strategy subscale of DERS; DERS-N: Nonacceptance subscale of DERS; DERS-A: Non-Awareness subscale of DERS; DERS-T: Total scores of DERS; Nona: Nonacceptance subscale of DERS-16;

DEP: Difficulties in Emotion Processing; DER: Difficulties in Emotional Response. *p < 0.05, **p < 0.01

**Table 3:** Correlations between subscales of the DERS-16, TAS, and BIS-11

	Clarity (1)	Goal (2)	Imp (3)	Stra (4)	Nona (5)	Total (6)	DEP (7)	DER (8)	TAS (9)	BIS11 (10)
1	1									
2	0.42**	1								
3	0.50**	0.42**	1							
4	0.61**	0.57**	0.60**	1						
5	0.42**	0.28**	0.42**	0.57**	1					
6	0.72**	0.71**	0.77**	0.91**	0.69**	1				
7	0.78**	0.40**	0.54**	0.69**	0.90**	0.83**	1			
8	0.63**	0.77**	0.79**	0.92**	0.53**	0.97**	0.67**	1		
9	0.70**	0.47**	0.50**	0.67**	0.53**	0.74**	0.71**	0.67**	1	
10	0.31**	0.33**	0.33**	0.33**	0.25**	0.40**	0.33**	0.39**	0.40**	1

Note. Imp: Impulsivity subscale of DERS-16; Stra: Strategy subscale of DERS-16; Nona: Nonacceptance subscale of DERS-16; DEP: Difficulties in Emotion Processing; DER: Difficulties in Emotional Regulation; TAS: Toronto Alexithymia Scale; BIS-11: Barrett Impulsivity Scale., **p < 0.01

**Table 4:** Correlations between subscales of DERS–36, TAS, and BIS-11

	Clarity (1)	Goal (2)	Imp (3)	Stra (4)	Nona (5)	Nonaw (6)	Total (7)	TAS (8)	BIS-11 (9)
1	1								
2	0.38**	1							
3	0.60**	0.39**	1						
4	0.60**	0.51**	0.70**	1					
5	0.47**	0.27**	0.52**	0.58**	1				
6	0.33**	-0.04	0.18**.	0.17**	0.22**	1			
7	0.78**	0.61**	0.80**	0.88**	0.74**	0.38**	1		
8	0.79**	0.42**	0.60**	0.67	0.55**	0.34**	0.79**	1	
9	0.33**	0.31**	0.36**	0.36**	0.28**	0.28**	0.45**	0.40**	1

Note. Imp: Impulsivity subscale of DERS-16; Stra: Strategy subscale of DERS-16; Nona: Nonacceptance subscale of DERS-16; TAS: Toronto Alexithymia Scale; BIS-11: Barrett Impulsivity Scale., **p < 0.01

## Discussion

In our study, the validity and reliability of the DERS-16 in Turkish adolescents (aged 14–18) were assessed for the first time. CFA was performed on the five-factor, single-factor, and second-order bifactor models. It was found that the CFA values of the single-factor model showed a poor fit, but the fit of both the five-factor and second-order bifactor models was good [13]. In the second-order bifactor and five-factor models, the GFI value was below the acceptable threshold of 0.90, likely because this index is affected by the sample size [25]. Previous studies, that are based on neurobiological hypotheses for ER and that have investigated the factorial construct of DERS-16, have not included the Strategy subscale in their CFA, because this subscale reflects only self-efficacy [10]. On the other hand, the Strategy subscale is retained within the DER subfactor, and in these studies, this model provided a good fit. In our study, as in the study by Moreira et al. [26], it was concluded that the fit of the second-order bifactor model in this way was good and usable. In the next stage of our study, based on the five-factor and second-order bifactor models, we conducted reliability analysis. The reliability coefficient of the total score of the scale was within the accepted range and the range found in studies of the DERS-16 in other countries [8]. Unlike our study, the two-item Clarity scale was also included in the reliability analysis. There is controversy in the literature regarding the reliability of two-item factors. While some authors argue that it is pointless to assess the reliability of two items, some authors reported the validity coefficients of both items [27, 28]. When reporting the reliability of a two-item scale, Eisanga et al. [29] suggest giving the Cronbach’s alpha value together with the Spearman-Brown coefficient. In our study, we would point out that the other subscales of the DERS-16 scale, apart from the Nonacceptance factor, were within reliable limits. Although the acceptable cut-off value of a reliable questionnaire is typically 0.70 in the literature [30], values of 0.60 or above may be accepted for short scales [31]. Studies of the DERS-16 scale in other countries have shown that the reliability values of the subscales are also within acceptable limits [32]. In the current study, the reliability of subscales and total scores of DERS-16 were in line with those reported in previous studies; the reliability values of second-order bifactor models were also within acceptable limits.

When the correlations between the subscales and subfactors of DERS-16 were examined, these varied between 0.28 and 0.91. In the study of Kaufman et al. [16], these varied between 0.04 and 0.85. In addition, correlations between the subscales of DERS-36 were in the same range. Furthermore, the correlations between the DERS-16 subscales and DERS-36 subscales ranged between 0.75 and 0.84, higher than Cohen’s recommended thresholds [33]. In the literature, it has been assumed that the high level of correlation between the short and long forms is a finding that indicates the validity of the short form [34]. In addition, the correlations between the DERS-16 and the TAS and BIS-11, and the correlations between the DERS-36 and these other scales, were at a similar level. This suggests that the construct validity of the DERS-16 was similar to that of the DERS-36.

To investigate the convergent validity of DERS-16, the correlations between the DERS-16, BIS-11, and TAS were analyzed. Considering the correlations between the TAS and the DERS subscales, the highest was found with the Clarity subscale, followed by the Strategy and then the Nonacceptance subscales. Studies showing that alexithymia is associated with both emotional processing and dysfunctional ER strategies support the high correlation between the total alexithymia score and the DERS Clarity, Nonacceptance, and Strategy subscales [35]. There was also a moderate correlation between the Impulsivity and Goal subscales. When an individual is exposed to a negative stimulus and is not able to understand and express the elicited feelings, the individual may remain uncertain, and to avoid this, may decide to act without thinking. Studies have shown that alexithymia is associated with goal-directed behavior and impulsivity [36]. When the relationship between the DERS-16 scale and the BIS-11 scale was examined, it was found that the BIS-11 had correlations with the DERS-16 subscales that ranged between 0.31 and 0.20, in line with the literature [36].

### Limitations

The present study had some limitations. First, the fact that the sample was accessed online may have reduced the generalizability of the findings, because adolescents of lower socioeconomic status and who do not have internet access might be underrepresented. Second, completing both the DERS-16 and DERS-36 scales in the same form may have decreased the motivation of the participants to read the scale, because some questions were repeated. Third, a scale measuring psychopathology was not included in our study, which already had many questions. Although the content validity of the DERS-16 scale was investigated by analyzing its relationships with the BIS-11 and TAS scales, the use of a scale measuring psychiatric symptoms might have strengthened our reported content validity. Finally, in the future, administering the scale with a larger and more representative (e.g., at the socioeconomic level) sample of adolescents will increase the generalizability of the findings.

## Conclusion

The current study shows that the DERS-16 scale is not statistically different from its original form in terms of scale structure and has satisfactory validity and reliability. In addition to this, it was revealed that the second-order bifactor model of DERS-16 was applicable. The fact that the DERS-16 scale has fewer items while retaining similar levels of reliability and validity, and that it can be calculated as a two-factor solution, provides advantages in terms of applicability.

## Data Availability

The datasets generated during and/or analyzed during the current study are available from the corresponding author on reasonable request.
